# A Genome-Wide Meta-Analysis of Six Type 1 Diabetes Cohorts Identifies Multiple Associated Loci

**DOI:** 10.1371/journal.pgen.1002293

**Published:** 2011-09-29

**Authors:** Jonathan P. Bradfield, Hui-Qi Qu, Kai Wang, Haitao Zhang, Patrick M. Sleiman, Cecilia E. Kim, Frank D. Mentch, Haijun Qiu, Joseph T. Glessner, Kelly A. Thomas, Edward C. Frackelton, Rosetta M. Chiavacci, Marcin Imielinski, Dimitri S. Monos, Rahul Pandey, Marina Bakay, Struan F. A. Grant, Constantin Polychronakos, Hakon Hakonarson

**Affiliations:** 1 The Center for Applied Genomics, The Children's Hospital Philadelphia, Philadelphia, Pennsylvania, United States of America; 2 Departments of Pediatrics and Human Genetics, McGill University, Montreal, Canada; 3 Department of Pediatrics, University of Pennsylvania School of Medicine, Philadelphia, Pennsylvania, United States of America; 4 Department of Pathology and Laboratory Medicine, Abramson Research Center, The Children's Hospital of Philadelphia, Philadelphia, Pennsylvania, United States of America; 5 Division of Human Genetics, Abramson Research Center, The Children's Hospital of Philadelphia, Philadelphia, Pennsylvania, United States of America; University of Oxford, United Kingdom

## Abstract

Diabetes impacts approximately 200 million people worldwide, of whom approximately 10% are affected by type 1 diabetes (T1D). The application of genome-wide association studies (GWAS) has robustly revealed dozens of genetic contributors to the pathogenesis of T1D, with the most recent meta-analysis identifying in excess of 40 loci. To identify additional genetic loci for T1D susceptibility, we examined associations in the largest meta-analysis to date between the disease and ∼2.54 million SNPs in a combined cohort of 9,934 cases and 16,956 controls. Targeted follow-up of 53 SNPs in 1,120 affected trios uncovered three new loci associated with T1D that reached genome-wide significance. The most significantly associated SNP (rs539514, *P* = 5.66×10^−11^) resides in an intronic region of the *LMO7* (LIM domain only 7) gene on 13q22. The second most significantly associated SNP (rs478222, *P* = 3.50×10^−9^) resides in an intronic region of the *EFR3B* (protein EFR3 homolog B) gene on 2p23; however, the region of linkage disequilibrium is approximately 800 kb and harbors additional multiple genes, including *NCOA1, C2orf79, CENPO, ADCY3, DNAJC27, POMC*, and *DNMT3A*. The third most significantly associated SNP (rs924043, *P* = 8.06×10^−9^) lies in an intergenic region on 6q27, where the region of association is approximately 900 kb and harbors multiple genes including *WDR27, C6orf120, PHF10, TCTE3, C6orf208, LOC154449, DLL1, FAM120B, PSMB1, TBP*, and *PCD2.* These latest associated regions add to the growing repertoire of gene networks predisposing to T1D.

## Author Summary

Despite the fact that there is clearly a large genetic component to type 1 diabetes (T1D), uncovering the genes contributing to this disease has proven challenging. However, in the past three years there has been relatively major progress in this regard, with advances in genetic screening technologies allowing investigators to scan the genome for variants conferring risk for disease without prior hypotheses. Such genome-wide association studies have revealed multiple regions of the genome to be robustly and consistently associated with T1D. More recent findings have been a consequence of combining of multiple datasets from independent investigators in meta-analyses, which have more power to pick up additional variants contributing to the trait. In the current study, we describe the largest meta-analysis of T1D genome-wide genotyped datasets to date, which combines six large studies. As a consequence, we have uncovered three new signals residing at the chromosomal locations 13q22, 2p23, and 6q27, which went on to be replicated in independent sample sets. These latest associated regions add to the growing repertoire of gene networks predisposing to T1D.

## Introduction

Diabetes impacts approximately 200 million people worldwide [1], with microvascular and cardiovascular disease being the primary complications. Approximately 10% of cases are type 1 diabetes (T1D) sufferers, with ∼3% increase in the incidence of T1D globally per year [2]. It is expected that the incidence is 40% higher in 2010 than in 1998 [3].

T1D is a clear example of a complex trait that results from the interplay between environmental and genetic factors. There are many lines of evidence that there is a strong genetic component to T1D, primarily due to the fact that T1D has high concordance among monozygotic twins [4] and runs strongly in families, together with a high sibling risk [5].

Prior to the era of GWAS, only five loci had been fully established to be associated with T1D. However, the majority of the other reported associations in the pre-GWAS era [6]–[8] remain highly doubtful, where an initial report of association does not hold up in subsequent replication attempts by other investigative groups. This previous hazy picture of the genetics of T1D can be put down to the use of the only methodologies that were available at the time and which were much more limited than GWAS i.e. the candidate gene approach (where genomic regions were studied based on biological reasoning) and family-based linkage methodologies. Inconsistent findings can also be attributed to small sample sizes i.e. when power is low the false discovery rate tends to be high; GWAS *per se* has not improved consistency, rather it has leveraged large, well powered sample sizes combined with sound statistical analyses.

It has been long established that approximately half of the genetic risk for T1D is conferred by the genomic region harboring the HLA class II genes (primarily *HLA-DRB1*, *-DQA1* and *-DQB1* genes), which encode the highly polymorphic antigen-presenting proteins. Other established loci prior to the application of GWAS are the genes encoding insulin (*INS*) [9]-[12], cytotoxic T-lymphocyte-associated protein 4 (*CTLA4*) [13]–[16], protein tyrosine phosphatase, non-receptor type 22 (*PTPN22*) gene [17], [18], interleukin 2 receptor alpha (*IL2RA*) [19]–[21] and ubiquitin-associated and SH3 domain-containing protein A (*UBASH3A*) [22].

The application of genome wide association studies (GWAS) has robustly revealed dozens of genetic contributors to T1D [23]–[29], the results of which have largely been independently replicated [30]–[36]. The most recently reported meta-analysis of this trait identified in excess of forty loci [29], including 18 novel regions plus confirmation of a number of loci uncovered through cross-disease comparisons [34]–[36]. As such, the risks conferred by these additional loci are relatively modest compared to the ‘low-hanging fruit’ described in the first studies and could only be ultimately uncovered when larger sample sizes were utilized.

We sought to expand further on this mode of analysis by combining our cohort with all publically released genome wide SNP datasets to identify additional loci contributing to the etiology of T1D. Unfortunately, there is a relative paucity of control genotype data in these publically available sources. To circumvent this problem, we combined individual level data from each available cohort and we then compared the cases with controls from two sources. We next separated all the individual level data into two groups, characterized by the type of genotyping platform that was used to genotype the samples, which would later be recombined using inverse-variance meta-analysis. The 6,523 cases genotyped on an Illumina BeadChip included subjects from McGill University, The Children's Hospital of Philadelphia (CHOP), The Diabetes Control and Complications Trial – Epidemiology of Diabetes Interventions and Complications (DCCT-EDIC) cohort, and the Type 1 Diabetes Genetics Consortium (T1DGC), which in turn were compared with 6,648 similarly genotyped controls recruited at CHOP. The 3,411 cases genotyped on Affymetrix arrays included subjects from the Genetics of Kidneys in Diabetes Study (GoKinD) and the Wellcome Trust Cases Control Consortium (WTCCC) that were then compared with 10,308 similarly genotyped controls, including being derived from non-autoimmune disease related cases from the WTCCC, as well as from the British 1958 Birth Cohort and the UK National Blood Service [24].

## Results

We compared the power of our meta-analysis to that of the previous largest meta-analysis to date. We have more than double the power of the Barrett et al. meta-analysis to find variants with a relative risk of 1.2 and approximately three times the power to detect variants with a relative risk of 1.1 [29] (Figure S1).

**Figure 1:**
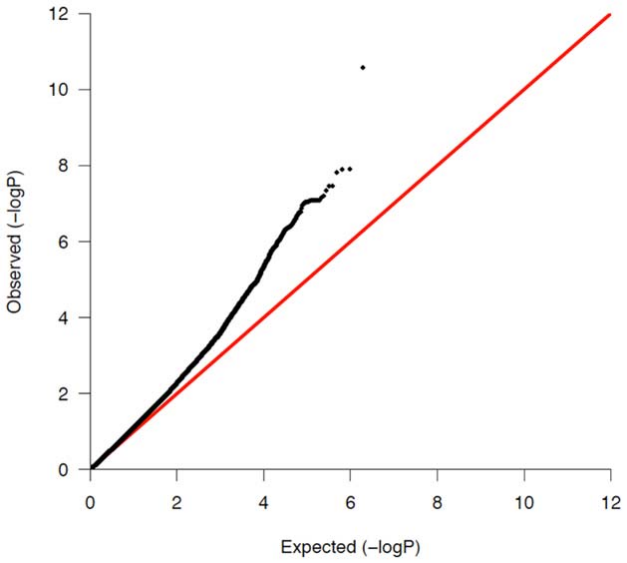
QQ-plot of all previously unassociated regions in the combined meta-analysis discovery cohort.

**Figure 2:**
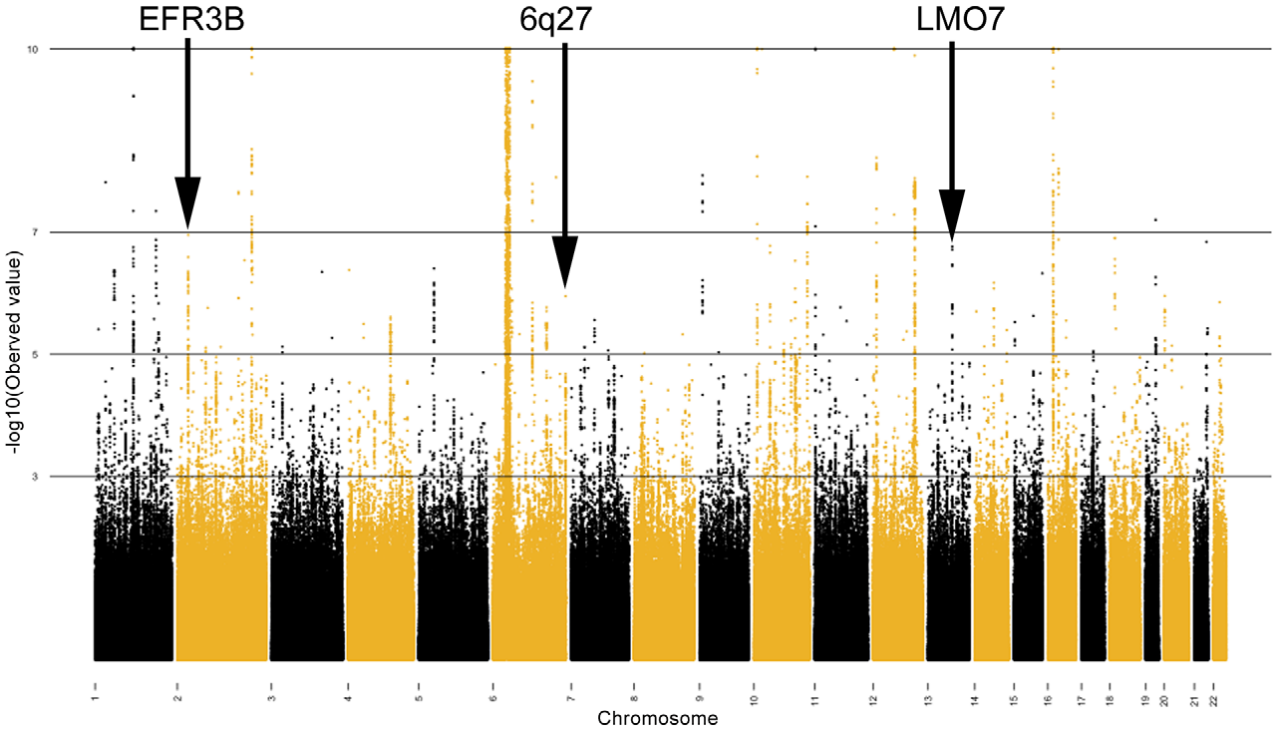
Fixed effects meta-analysis *P*-values shown for each SNP in the combined meta-analyzed discovery cohort. SNPs are sorted by chromosomal location. –log10(*P*-value) are shown, where the minimum *P*-value has been capped at 1×10^−10^. Only the novel loci are indicated.

We used principal components analysis (PCA) [37] in order to minimize the potential impact of population stratification in our case/control sample sets. Eigenstrat 3.0 was employed to remove outliers and to subsequently calculate the principal components in the Illumina and Affymetrix assigned groups separately. The principal components were then used as covariates in a logistic regression, using the software PLINK [38], to compute the *P*-values, betas and standard errors which were combined in our fixed effects inverse variance meta-analysis. After controlling for population stratification, the λ in the Affymetrix and Illumina cohorts was 1.11 and 1.17, respectively (see Figure 1 for Q-Q plot). A full description of the correlation of each eigenvector with known continental ancestry appears in Text S1. Mach was used to impute ∼2.54 million SNPs, including HapMap Phase 2 SNPs in the Illumina and Affymetrix datasets in order for the statistics to be uniform and amenable to being combined [39]. Results from the meta-analysis of this resulting ‘discovery’ cohort are shown Table 1 and graphically in Figure 2.

**Table 1:** SNPs are shown with *P*<0.05 in the replication set.

					Minor Allele	Affymetrix		Illumina		Meta-Analysis		Replication		Combined
						n = 13,719		n = 13,171				n = 1120		
SNP	Chr	Position	Gene/Region	Minor Allele	Frequency	OR	*P*	OR	*P*	OR	*P*	OR	*P*	*P*
Achieved GW significance overall														
rs539514	13	75224283	*LMO7*	A	0.499	0.8801	2.91×10^−4^	0.8769	1.65×10^−4^	0.8785	1.74×10^−7^	0.7048	1.16×10^−5^	5.66×10^−11^
rs478222	2	25155259	*EFR3B*	T	0.412	0.8838	7.39×10^−4^	0.8632	3.79×10^−5^	0.8732	1.12×10^−7^	0.818	1.32×10^−3^	3.50×10^−9^
rs924043	6	170220950	6q27	T	0.146	0.8979	4.44×10^−2^	0.7822	1.50×10^−6^	0.8353	1.12×10^−6^	0.7352	3.16×10^−4^	8.06×10^−9^
Did not achieve GW significance overall														
rs550448	7	28195567	*LOC100128081*	G	0.143	0.8792	1.31×10^−2^	0.8139	1.12×10^−4^	0.8468	7.70×10^−6^	0.7621	3.29×10^−3^	4.68×10^−7^
rs12679857	8	120046518	*TNFRSF11B*	G	0.309	0.9204	3.35×10^−2^	0.8465	1.50×10^−5^	0.8822	4.76×10^−6^	0.83	4.71×10^−3^	4.17×10^−7^
rs6547853	2	28500305	*FOSL2*	A	0.403	0.9302	4.76×10^−2^	0.8567	1.49×10^−5^	0.8919	7.40×10^−6^	0.8383	5.67×10^−3^	7.54×10^−7^

All *P*-values are two-sided. All odds ratios are shown with respect to the minor allele. Combined *P*-values were computed with Fishers combined *P*-value technique implemented in HaploView. Loci reported for the first time in this current study are annotated. Positions shown are based on Build 36 of the human genome. Minor allele frequencies are shown for the controls in the discovery cohort.

53 SNPs were brought forward to the replication stage based on satisfying the following criteria; however one of these SNPs consistently failed genotyping in the replication effort. The most significantly associated SNP at a given locus if the meta-analysis *P*-value was less than 1×10^−5^ (an independent locus was defined as a region for a given focal SNP, where we extended the region in both directions until either 250 kb had been traversed or until reaching another SNP with *P*<10^−5^), the Cochran's Q statistic *P*-value was greater than 0.05 and the locus had not been already reported from a previous GWAS of T1D. A table outlining the results for all previously described T1D associated SNPs plus our strongest associations for known regions associated with the disease are shown in Table 2 and Table S1, respectively. The replication cohort consisted of additional T1D affected trios from the T1DGC and McGill which had not been part of the original discovery cohort. The replication cohort was genotyped using the Sequenom iPLEX system and the results were analyzed using the transmission disequilibrium test in PLINK. Results for both the discovery and replication cohorts for the six SNPs that replicated with *P*≤0.05 are shown in Table 1 (the full outcomes for all SNPs tested are in Table S2).

**Table 2:** Discovery set *P*-values and odd ratios are shown for known T1D associated autosomal SNPs.

SNP	CHR	Position	Gene/Region	Effect Allele	*P*-Value	OR	References
rs2476601	1	114179091	*PTPN22*	A	5.93E-80	1.96	[23], [25], [29]
rs2816316	1	190803436	*RGS1*	C	8.52E-04	0.89	[34]
rs3024505	1	205006527	*IL10*	A	2.09E-08	0.82	[29]
rs9653442	2	100191799	*AFF3*	C	5.89E-04	1.09	[25]
rs1990760	2	162832297	*IFIH1*	C	2.21E-08	0.87	[25], [29]
rs7574865	2	191672878	*STAT4*	T	0.0544	1.06	[35]
rs3087243	2	204447164	*CTLA4*	A	1.42E-13	0.83	[27], [29]
rs11711054	3	46320615	*CCR5*	G	0.0399	1.06	[34]
rs10517086	4	25694609	*4p15.2*	A	2.13E-04	1.10	[29]
rs4505848	4	123351942	*IL2*	G	2.26E-05	1.12	[27], [29]
rs9268645	6	32516505	*HLA*	G	3.94E-136	1.91	[24]
rs3757247	6	91014184	*BACH2*	T	1.62E-08	1.15	[27]-[29]
rs9388489	6	126740412	*6q22.32*	G	4.10E-06	1.12	[29]
rs10499194	6	138044330	*TNFAIP3*	T	7.92E-04	0.91	[35]
rs1738074	6	159385965	*TAGAP*	T	9.48E-05	0.91	[34]
rs7804356	7	26858190	*SKAP2*	C	0.0101	0.93	[29]
rs4948088	7	50994688	*7p12.1*	NA	NA	NA	[29]
rs10758593	9	4282083	*GLIS3*	A	1.18E-08	1.15	[28], [29]
rs12251307	10	6163501	*IL2RA*	T	1.22E-08	0.79	[27], [29]
rs11258747	10	6512897	*PRKCQ*	T	2.24E-05	1.13	[27], [29]
rs10509540	10	90013013	*10q23.31*	C	2.83E-06	0.88	[29]
rs3741208	11	2126350	*INS*	A	6.33E-08	1.16	[23], [25], [29]
rs4763879	12	9801431	*12p13.31*	A	6.45E-07	1.14	[24], [29]
rs1701704	12	54698754	*12q13.2*	G	1.08E-30	1.35	[24]-[27], [29]
rs10877012	12	56448352	*CYP27B1*	NA	NA	NA	[54]
rs3184504	12	110368991	*SH2B3*	C	1.77E-21	0.79	[29]
rs9585056	13	98879767	*GPR183*	C	1.27E-03	1.09	[55]
rs1465788	14	68333352	*14q24.1*	T	1.79E-06	0.87	[29]
rs4900384	14	97568704	*14q32.2*	G	0.0972	1.05	[29]
rs941576	14	100375798	*DLK1*	G	9.33E-05	0.91	[56]
rs17574546	15	36689768	*RASGRP1*	C	3.19E-03	1.09	[57]
rs3825932	15	77022501	*15q25.1*	T	5.15E-05	0.90	[27], [29]
rs2903692	16	11146284	*16p13.13*	A	4.21E-15	0.81	[23]-[25], [27], [29]
rs4788084	16	28447349	*IL27*	T	7.55E-04	0.92	[29]
rs7202877	16	73804746	*16q23.1*	G	1.84E-05	1.19	[29]
rs2290400	17	35319766	*ORMDL3*	T	3.55E-03	0.93	[29]
rs7221109	17	36023812	*17q21.2*	T	6.46E-04	0.92	[29]
rs478582	18	12825976	*PTPN2*	C	7.72E-04	0.92	[24], [25], [27], [29]
rs763361	18	65682622	*CD226*	T	1.17E-04	1.10	[25]
rs2304256	19	10336652	*TYK2*	NA	NA	NA	[56]
rs425105	19	51900321	*19q13.32*	C	5.51E-06	0.85	[29]
rs2281808	20	1558551	*20p13*	T	2.06E-06	0.88	[29]
rs9976767	21	42709459	*UBASH3A*	G	1.69E-05	1.11	[28], [29]
rs5753037	22	28911722	*22q12.2*	T	0.0164	1.06	[29]
rs229541	22	35921264	*IL2RB*	A	3.67E-06	1.12	[27], [29]

The list of known SNPs was collected from references cited in the references column and shown below. One SNP from each locus was chosen when multiple SNPs from the same locus are known. NA in the effect allele, *P*-value, and OR column refers to SNPs that were not imputed in the discovery cohort. Positions shown are based on Build 36 of the human genome.

We combined the ‘discovery’ and ‘replication’ meta-analysis *P*-values using Fisher's combined *P*-value method implemented in Haploview, comparable to what has been previously employed by others [40]. Three of the SNPs, namely rs539514, rs478222 and rs924043, had combined *P*-values <5×10^−8^, the statistical threshold for genome wide significance, while the remaining three, namely rs550448, rs12679857 and rs6547853, failed to reach this bar but were suggestive of association as the alleles yielded both a consistent direction of effect and *P*-values <0.05 in the replication cohort. These two categories of outcome are summarized in Table 1; in addition, these six SNPs were further investigated with respect to adjustments of the discovery and met-analysis *P*-values based on the lambdas of each respective cohort (Table S3).

## Discussion

We have carried out the largest meta-analysis of genome wide genotyped datasets for T1D to date. The replication of three loci using the stratification-free TDT with minimal Mendelian error clearly indicates that they are not false positives due to artifacts such as uncorrected systematic error from stratification or genotyping bias.

The most significantly associated SNP (rs539514, *P* = 5.66×10^−11^) resides in an intronic region of the *LMO7* (LIM domain only 7) gene on 13q22. We investigated the associated region using LocusZoom [41] and determined that it is the only gene residing within the block of linkage disequilibrium harboring the signal (Figure S3). Regional plots showing *P*-values, linkage disequilibrium, and recombination rate for all SNPs in Table 1 are outlined in the Figures S2, S3, S4, S5, S6, S7. *LMO7* encodes a protein that contains multiple domains, including a calponin homology domain, a PDZ domain and a LIM domain. There are multiple LMO7 isoforms already known but their full nature and the actual extent of different isoforms remains unclear [42]. Mice with homozygous deletions of *LMO7* display retinal, muscular, and growth retardation [43]. Although the function of LMO7 doesn't clearly relate to the etiology of T1D, *LMO7* is expressed in pancreatic islets and thus is a possible biological candidate at this locus [44]; however it should be noted that the retinal, muscular development and islet patterns are a key element in Emery-Dreifuss Muscular Dystrophy, caused by mutations in *LMO7* [45], but bears very little similarity to T1D.

The second most significantly associated SNP (rs478222, *P* = 3.50×10^−9^) resides in an intronic region of the *EFR3B* (protein EFR3 homolog B) gene on 2p23; however the region of linkage disequilibrium is approximately 800 kb and harbors additional multiple genes, including 3*NCOA1, C2orf79, CENPO, ADCY3, DNAJC27, POMC*, and *DNMT3A*. (Figure S2). A previous meta-analysis of a subset of the data used in this current study found suggestive association with T1D in the same LD block with the independent SNP, rs2165738(r^2^ = 0.115), but did not achieve genome wide significance at that time (*P* = 3.65×10^−6^) [27]; however, we only found modest evidence of association with rs2165738 (*P* = 4.78×10^−3^) in our discovery cohort. There has also been association to inflammatory bowel disease [46] height [47], [48] and BMI [49] reported at this locus, where in both cases the risk allele for increased height or BMI was protective for T1D risk.

The third most significantly associated SNP (rs924043, *P* = 8.06×10^−9^) lies in an intergenic region on 6q27, where the region of association is approximately 900 kb and harbors multiple genes including *WDR27, C6orf120, PHF10, TCTE3, C6orf208, LOC154449, DLL1, FAM120B, PSMB1, TBP* and *PCD2* (Figure S5). In addition, despite not reaching the bar for genome wide significance, we did observe evidence for association at three additional loci (Table 1) containing the candidate genes *LOC100128081*, *TNFRSF11B* and *FOSL2.* Of these, it is notable that *TNFRSF11B* is a strongly associated locus with bone mineral density, also as a consequence of GWAS [50], [51]. In addition, the locus harboring *LOC100128081* has also been reported in the context of a GWAS of SLE [52]. Further work will be required to fully validate the role of these particular loci in the pathogenesis of T1D.

The Barrett *et al*. meta-analysis was able to use British controls with British cases and American controls with American cases [29]. We did not have the same control data to be able to make the same comparisons. In the case of the Affymetrix analysis, some American cases were analyzed with purely British controls and, in the case of the Illumina analysis, some British cases with purely American controls. As such, we were forced to make our corrections using eigenvectors as covariates in our analysis; this will have the effect of modestly weakening the level of significance for associations that vary in allele frequency between the cases and controls, as now the case and controls will both vary with the eigenvectors to some degree. This in effect will make our analysis overly conservative with estimating the true effect of a SNP, and in fact every SNP that had a *P*-value less than 0.05 in the replication set did indeed have a greater effect than that which was estimated from the discovery set.

In summary, we provide convincing evidence for the existence of three additional loci associated with the T1D, adding to the repertoire of over 50 loci already demonstrated to be associated with the disease.

## Materials and Methods

### Ethical statement

The study was approved by the institutional review board and the ethics committee of each institution. Written informed consent was obtained from each participant in accordance with institutional requirements and the Declaration of Helsinki Principles.

### Samples

Cases in the discovery set were obtained from four publically available resources and combined with those from a previous publication for the meta-analysis. Samples descriptions are available on dbgap (http://www.ncbi.nlm.nih.gov/sites/entrez?db=gap) for the T1DGC (http://www.ncbi.nlm.nih.gov/projects/gap/cgi-bin/study.cgi?study_id=phs000180.v1.p1), GoKinD (http://www.ncbi.nlm.nih.gov/projects/gap/cgi-bin/study.cgi?study_id=phs000088.v1.p1), and DCCT-EDIC (http://www.ncbi.nlm.nih.gov/projects/gap/cgi-bin/study.cgi?study_id=phs000086.v2.p1) patients. The WTCCC sample information is available from [24]. Samples from the T1D segment of the WTCCC were used as cases, while controls were derived from the 1958 Birth Cohort, UK Blood Service, Bipolar disorder, Coronary heart disease, Hypertension, and Type 2 Diabetes segments. The remaining cases used in the meta-analysis were previously described [23].

The total number of individuals used in the meta-analysis discovery set was 26,890 (9,934 cases/16,956 controls). The replication set consisted of 1120 case-parent trios from the T1DGC and those identified through pediatric diabetes clinics in Canada. The replication set was identical to that used in Hakonarson et al. with an extension of patients identified through pediatric diabetes clinics in Montreal, Toronto, Ottawa, and Winnipeg. All individuals were of Caucasian ancestry. A breakdown of the number of samples in each cohort in the discovery phase and a comparison with the numbers used in the Barrett et al. meta-analysis are shown in Table 3 [29]. The minor variation in the number of cases reflects that, despite using slight differences in both quality control and methods for dealing with population stratification, we have comparable numbers of cases from this cohort remaining in our analysis. Primarily, this small difference is due to the fact that we strictly accounted for relatedness and duplicates within and across cohorts in this current setting.

**Table 3:** A comparison of the number of samples used in each discovery cohort from the current meta-analysis and those used in the previously reported meta-analysis [29] .

	WTCCC	GoKinD/NIMH	DCCT-EDIC	T1DGC	CHOP-McGill	Totals
**Cases in Barrett et al meta-analysis**	1,930	1,601	0	3,983	0	7,514
**Controls in Barrett et al meta-analysis**	3,342	1,704	0	3,999	0	9,045
**Cases in current meta-analysis**	1,920	1,491	1,363	4,029	1,131	9,934
**Controls in current meta-analysis**	10,308	0	0	0	6,648	16,956

### Power analysis

Power analysis was performed with the genetic analysis calculator which can be found at (http://pngu.mgh.harvard.edu/~purcell/gpc/) [53]. Various assumption were made included perfect LD between the causative variant and the markers that were genotyped, an additive genetic model, a disease prevalence of 0.0033 and an alpha of 1×10^−5^.

### Genotyping, quality control, and imputation

Discovery samples from Philadelphia, Canada, T1DGC, and DCCT-EDIC were genotyped on a mixture of the Illumina HumanHap 550v1, 2, and 3, whereas samples from GoKinD and WTCCC were genotyped on the Affymetrix 500 K Chip. Sequenom iPlex was used to replicate the findings of the meta-analysis in 1,120 affected offspring trios from the T1DGC and from Canada.

All individuals needed an individual genotyping call rate greater than 0.98 to be included in the analysis pre-imputation and individuals were removed that showed evidence of cryptic relatedness and duplication within and across cohorts using identity-by-state. SNP quality control was performed on all samples pre-imputation. SNPs were excluded from the analysis if the minor allele was below 1%, the genotyping call rate was less than 95%, or the Hardy Weinberg equilibrium *P*-value was less than 0.00001.

To control for population stratification, Eigenstrat 3.0 was used to compute the top 10 principal components of the individuals genotyped on the Illumina SNP chips and the Affymetrix SNP chips separately [37]. Individuals were removed from the analysis if they were 6 standard deviations away from the mean of one of the top 10 principal components. After controlling for population stratification, the estimated lambda in the Affymetrix data was 1.11 and 1.17 in the Illumina data.

Mach 1.0 was used to impute ∼2.54 millions SNPs from the HapMap CEU panel for all individuals [39]. SNPs were excluded after imputation if they had a minor allele frequency less than 0.01 and an r2 value less than 0.3.

### Genome-wide association and meta-analysis

PLINK [38] was used to perform a logistic regression using the 10 principal components as covariates, T1D status as the outcome, and in the case of the Affymetrix cohort, an extra dummy covariate specifying WTCCC or GoKinD cohort membership. Results from the logistic regression of 2,436,110 SNPs from the Affymetrix samples and 2,062,307 SNPs from the Illumina samples separately were combined using inverse-variance meta-analysis in PLINK. A fixed effects meta-analysis was performed and 53 SNPs were chosen for replication who had a fixed effects *P*-value <0.00001, a Cochran's Q statistic *P*-value greater than 0.05 and were not previously known to be associated with type 1 diabetes. However one of the SNPs consistently failed during the replication effort.

## Supporting Information

Figure S1 Comparison of plot of power for previous and current meta-analyses. a: Plot of power (y-axis) for variants from the previously reported meta-analysis [29] with various allele frequencies (x-axis) and relative risks. Plots assume disease prevalence of 0.0033, an additive genetic model, perfect LD between causative variant and marker, and are shown for an alpha of 1×10^−5^. b: Plot of power (y-axis) in the current meta-analysis for variants with various allele frequencies (x-axis) and relative risks. Plots assume disease prevalence of 0.0033, an additive genetic model, perfect LD between causative variant and marker, and are shown for an alpha of 1×10^−5^.
(DOC)

Figure S2 Regional plot of the *EFRB* associated region. –log10(*P*-values) are shown for all SNPs in the region and color of circles indicates degree of LD with the most associated SNP in the region. Recombination rate is overlaid on the figure and the position with respect to genes is shown at the bottom.
(DOC)

Figure S3 Regional plot of the *LMO7* associated region. –log10(*P*-values) are shown for all SNPs in the region and color of circles indicates degree of LD with the most associated SNP in the region. Recombination rate is overlaid on the figure and the position with respect to genes is shown at the bottom.
(DOC)

Figure S4 Regional plot of the *LOC100128081* associated region. –log10(*P*-values) are shown for all SNPs in the region and color of circles indicates degree of LD with the most associated SNP in the region. Recombination rate is overlaid on the figure and the position with respect to genes is shown at the bottom.
(DOC)

Figure S5 Regional plot of the Chromosome 6 associated region. –log10(*P*-values) are shown for all SNPs in the region and color of circles indicates degree of LD with the most associated SNP in the region. Recombination rate is overlaid on the figure and the position with respect to genes is shown at the bottom.
(DOC)

Figure S6 Regional plot of the Chromosome 8 associated region. –log10(*P*-values) are shown for all SNPs in the region and color of circles indicates degree of LD with the most associated SNP in the region. Recombination rate is overlaid on the figure and the position with respect to genes is shown at the bottom.
(DOC)

Figure S7 Regional plot of the Chromosome 2 *FOSL2* associated region. –log10(*P*-values) are shown for all SNPs in the region and color of circles indicates degree of LD with the most associated SNP in the region. Recombination rate is overlaid on the figure and the position with respect to genes is shown at the bottom.
(DOC)

Table S1 Discovery set *P*-values and odd ratios are shown for strongest associated SNP in known T1D associated regions. The list of known associated regions was collected from references cited in the references column and shown below.
(DOC)

Table S2 *P*-values and odds ratios of discovery and replication cohort are shown for all SNPs taken forward to replication stage. Combined *P*-values are shown for all SNPs that had the same direction of effect. *P*-values were combined using the Fishers combined *P*-value method implemented in Haploview. NA refers to a different direction of effect and the *P*-value was never computed. One SNP, rs722988, which failed in the genotyping assay is not shown.
(DOC)

Table S3 *P*-values for the six SNPs highlighted in Table 1 following adjustment for lambdas.
(DOC)

Text S1 Correlation outcomes of Eigenvectors with known continental ancestry.
(DOC)
