# Supplementary figures and images for: A Genome-Wide Meta-Analysis of Six Type 1 Diabetes Cohorts Identifies Multiple Associated Loci
Source: PLoS Genet. 2011 Sep 29;7(9):e1002293. doi: 10.1371/journal.pgen.1002293 (PMC3183083; doi:10.1371/journal.pgen.1002293)

**Figure S1a**


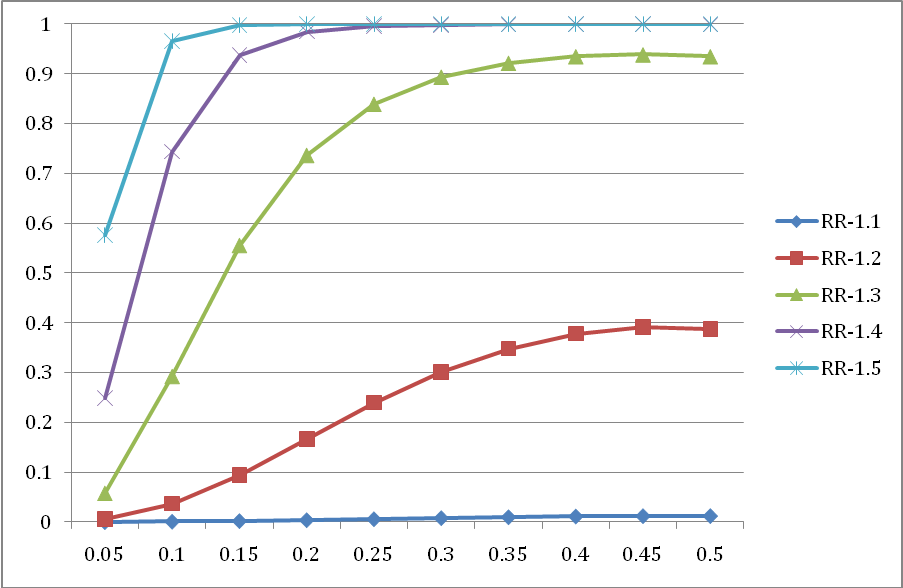


**Figure S1b**


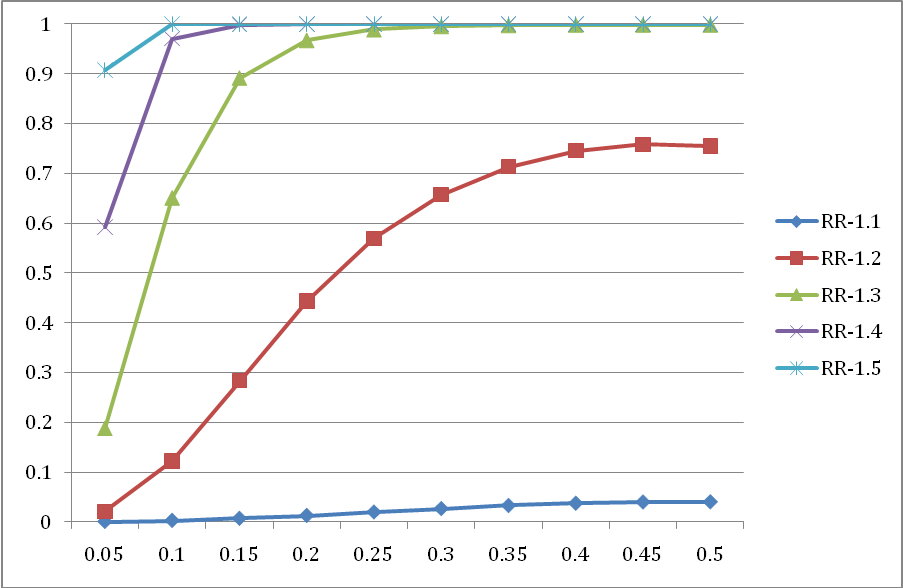

Supplement: Figure S1 — Comparison of plot of power for previous and current meta-analyses. a: Plot of power (y-axis) for variants from the previously reported meta-analysis [29] with various allele frequencies (x-axis) and relative risks. Plots assume disease prevalence of 0.0033, an additive genetic model, perfect LD between causative variant and marker, and are shown for an alpha of 1×10^−5^. b: Plot of power (y-axis) in the current meta-analysis for variants with various allele frequencies (x-axis) and relative risks. Plots assume disease prevalence of 0.0033, an additive genetic model, perfect LD between causative variant and marker, and are shown for an alpha of 1×10^−5^. (DOC) [file pgen.1002293.s001.doc]

**Figure S2**


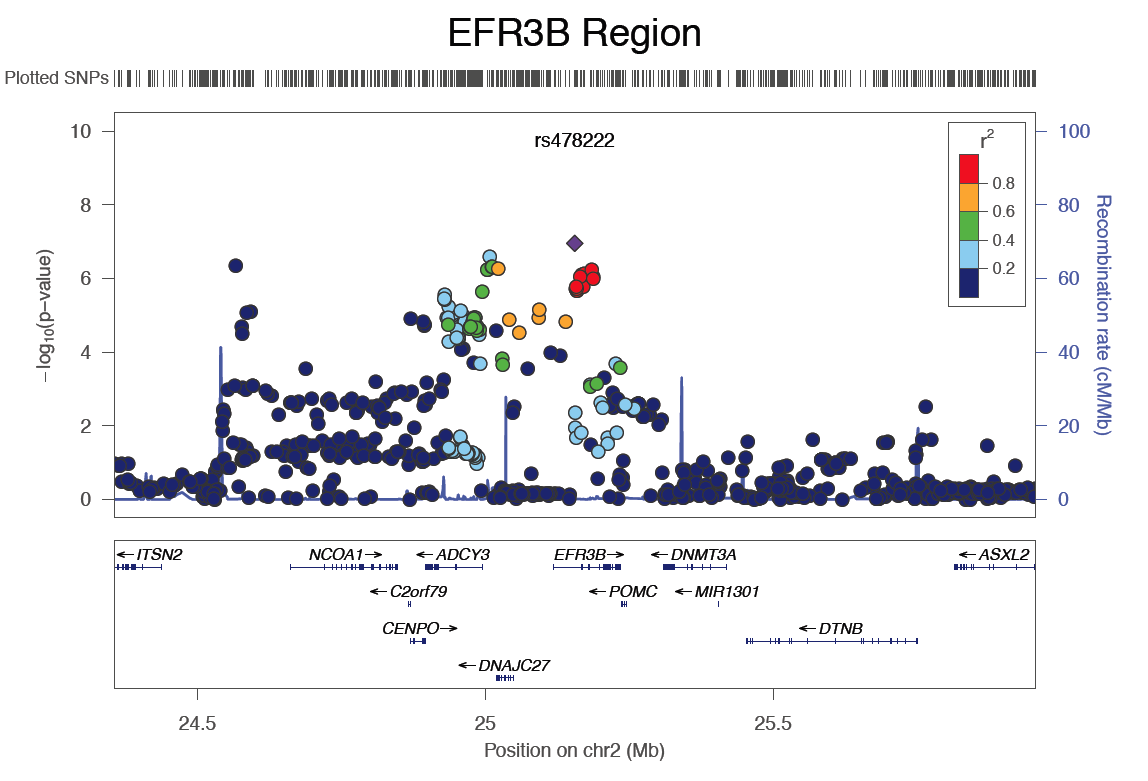

Supplement: Figure S2 — Regional plot of the EFRB associated region. –log10(P-values) are shown for all SNPs in the region and color of circles indicates degree of LD with the most associated SNP in the region. Recombination rate is overlaid on the figure and the position with respect to genes is shown at the bottom. (DOC) [file pgen.1002293.s002.doc]

**Figure S3**


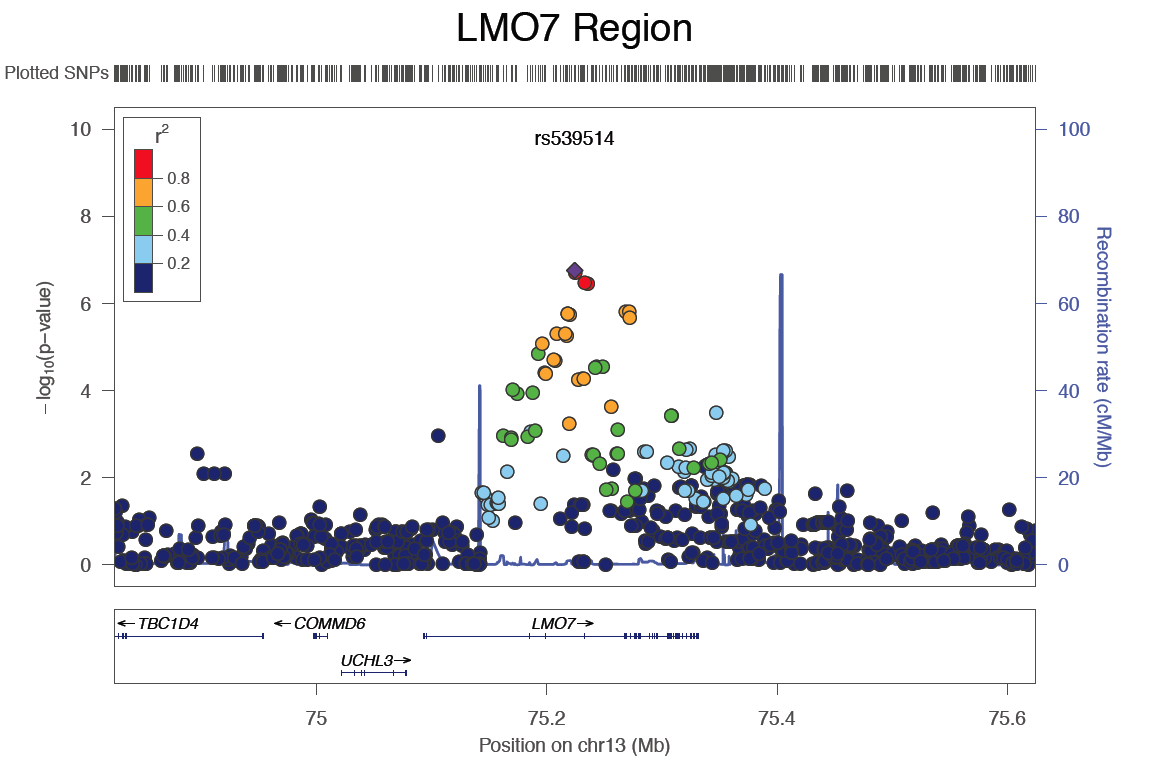

Supplement: Figure S3 — Regional plot of the LMO7 associated region. –log10(P-values) are shown for all SNPs in the region and color of circles indicates degree of LD with the most associated SNP in the region. Recombination rate is overlaid on the figure and the position with respect to genes is shown at the bottom. (DOC) [file pgen.1002293.s003.doc]

**Figure S4**


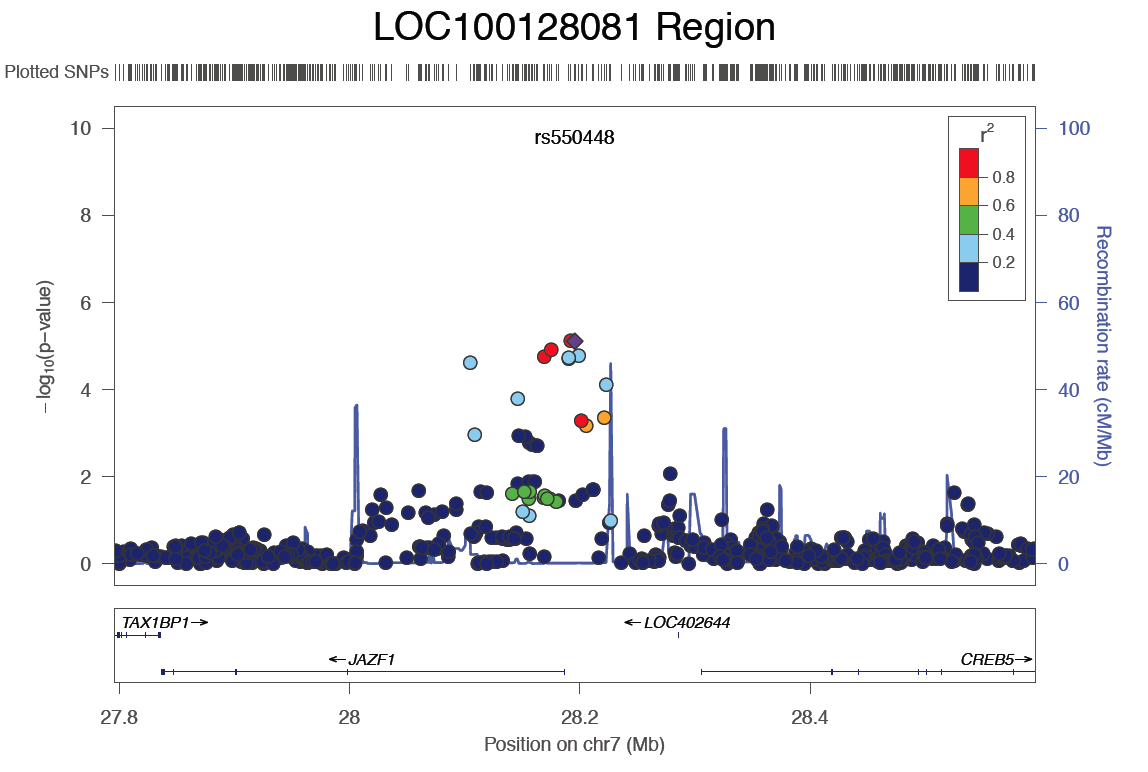

Supplement: Figure S4 — Regional plot of the LOC100128081 associated region. –log10(P-values) are shown for all SNPs in the region and color of circles indicates degree of LD with the most associated SNP in the region. Recombination rate is overlaid on the figure and the position with respect to genes is shown at the bottom. (DOC) [file pgen.1002293.s004.doc]

**Figure S5**


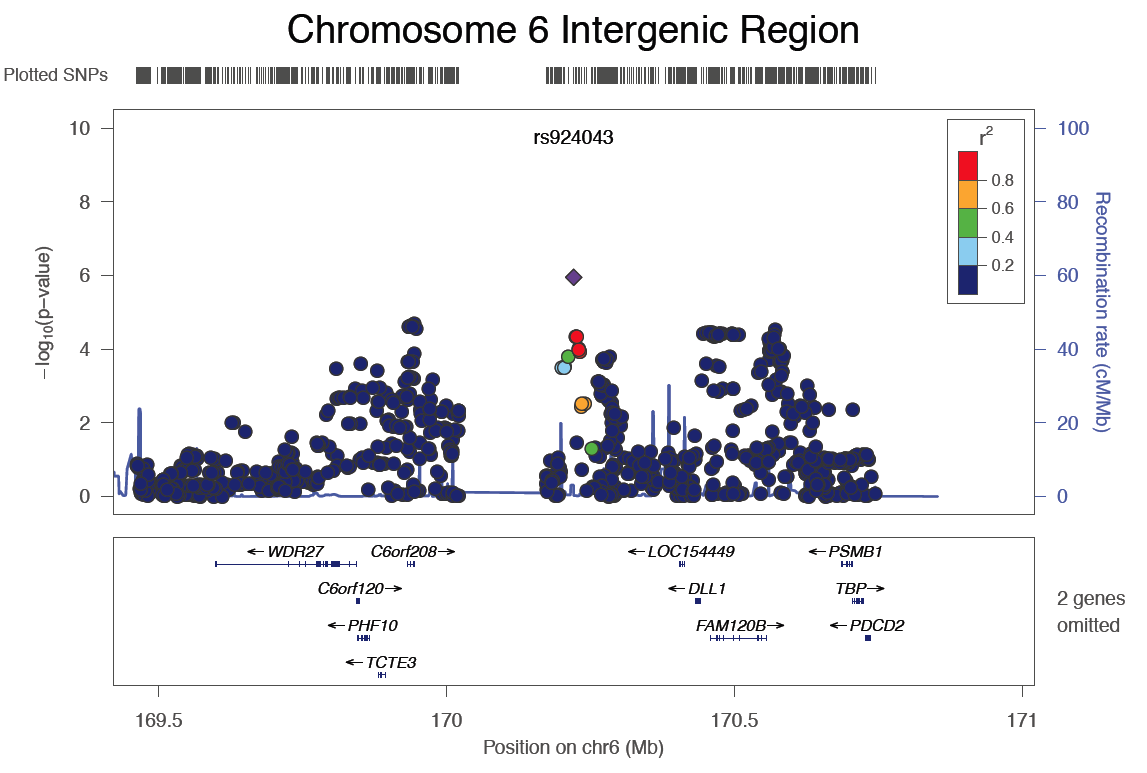

Supplement: Figure S5 — Regional plot of the Chromosome 6 associated region. –log10(P-values) are shown for all SNPs in the region and color of circles indicates degree of LD with the most associated SNP in the region. Recombination rate is overlaid on the figure and the position with respect to genes is shown at the bottom. (DOC) [file pgen.1002293.s005.doc]

**Figure S6**


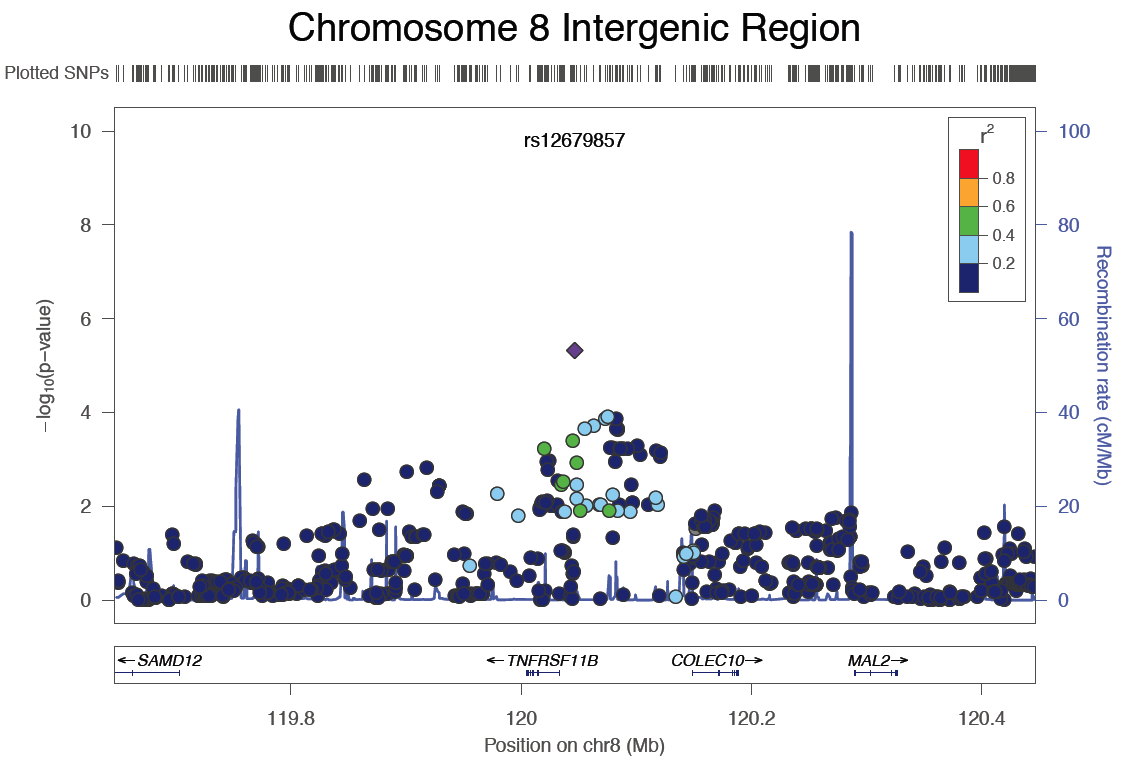

Supplement: Figure S6 — Regional plot of the Chromosome 8 associated region. –log10(P-values) are shown for all SNPs in the region and color of circles indicates degree of LD with the most associated SNP in the region. Recombination rate is overlaid on the figure and the position with respect to genes is shown at the bottom. (DOC) [file pgen.1002293.s006.doc]

**Figure S7**


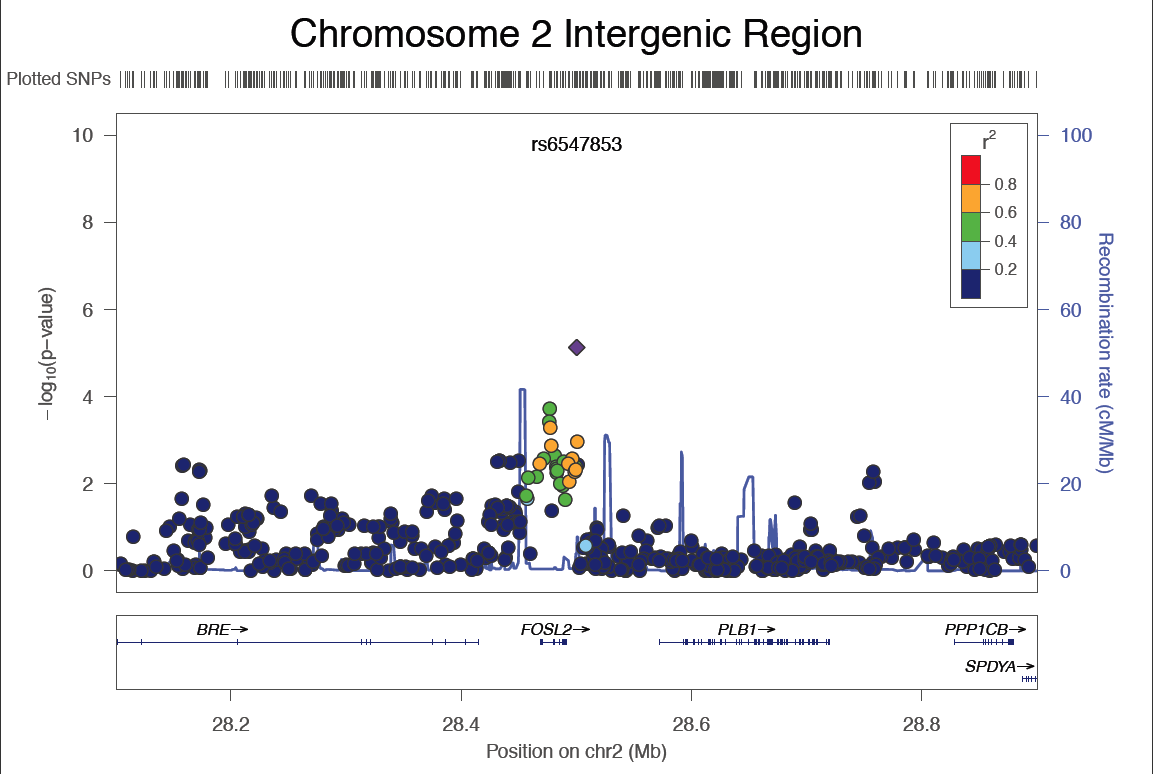

Supplement: Figure S7 — Regional plot of the Chromosome 2 FOSL2 associated region. –log10(P-values) are shown for all SNPs in the region and color of circles indicates degree of LD with the most associated SNP in the region. Recombination rate is overlaid on the figure and the position with respect to genes is shown at the bottom. (DOC) [file pgen.1002293.s007.doc]
