# Supplementary material for: A Genome-Wide Meta-Analysis of Six Type 1 Diabetes Cohorts Identifies Multiple Associated Loci
Source: PLoS Genet. 2011 Sep 29;7(9):e1002293. doi: 10.1371/journal.pgen.1002293 (PMC3183083; doi:10.1371/journal.pgen.1002293)
Supplement: Table S1 — Discovery set P-values and odd ratios are shown for strongest associated SNP in known T1D associated regions. The list of known associated regions was collected from references cited in the references column and shown below. (DOC) [file pgen.1002293.s008.doc]

**Table S1**

| **SNP** | **CHR** | **Position** | **Gene/Region** | **Effect Allele** | **P-Value** | **OR** | **References** |
| --- | --- | --- | --- | --- | --- | --- | --- |
|  |  |  |  |  |  |  |  |
| rs2476601 | 1 | 114179091 | *PTPN22* | A | 5.93E-80 | 1.96 | [1-3] |
| rs2209014 | 1 | 190772705 | *RGS1* | G | 2.16E-04 | 0.89 | [4] |
| rs3024493 | 1 | 205010591 | *IL10* | A | 2.01E-08 | 0.82 | [3] |
| rs6740838 | 2 | 100179931 | *AFF3* | T | 5.12E-06 | 1.12 | [1] |
| rs1990760 | 2 | 162832297 | *IFIH1* | C | 2.21E-08 | 0.87 | [1,3] |
| rs6744380 | 2 | 191709783 | *STAT4* | A | 1.22E-04 | 1.10 | [5] |
| rs11571316 | 2 | 204439334 | *CTLA4* | A | 2.44E-15 | 0.82 | [3,6] |
| rs17078977 | 3 | 46611534 | *CCR5* | T | 4.03E-04 | 0.87 | [4] |
| rs11933540 | 4 | 25729099 | *4p15.2* | C | 1.76E-04 | 1.10 | [3] |
| rs6827756 | 4 | 123403861 | *IL2* | T | 2.47E-06 | 1.13 | [3,6] |
| rs6916742 | 6 | 32561169 | *HLA* | T | <4.56E-307 | 0.24 | [7] |
| rs597325 | 6 | 91059215 | *BACH2* | A | 3.38E-10 | 0.85 | [3,6,8] |
| rs9375435 | 6 | 126703551 | *6q22.32* | T | 1.71E-06 | 1.13 | [3] |
| rs1878658 | 6 | 138020079 | *TNFAIP3* | G | 1.86E-04 | 0.88 | [5] |
| rs212402 | 6 | 159392283 | *TAGAP* | G | 2.11E-05 | 0.89 | [4] |
| rs12533947 | 7 | 27141820 | *SKAP2* | G | 7.08E-04 | 1.12 | [3] |
| rs10231420 | 7 | 51020309 | *7p12.1* | G | 2.76E-06 | 0.80 | [3] |
| rs10758593 | 9 | 4282083 | *GLIS3* | A | 1.18E-08 | 1.15 | [3,8] |
| rs7090530 | 10 | 6150881 | *IL2RA* | C | 2.93E-15 | 0.82 | [3,6] |
| rs2236380 | 10 | 6509823 | *PRKCQ* | A | 1.87E-06 | 1.14 | [3,6] |
| rs10509540 | 10 | 90013013 | *10q23.31* | C | 2.83E-06 | 0.88 | [3] |
| rs7928968 | 11 | 2006875 | *INS* | T | 2.78E-14 | 1.25 | [1-3] |
| rs10492166 | 12 | 9777266 | *12p13.31* | A | 6.04E-09 | 0.87 | [3,7] |
| rs705704 | 12 | 54721679 | *12q13.2* | A | 4.31E-31 | 1.35 | [1,3,6,7,9] |
| rs4760341 | 12 | 56567762 | *CYP27B1* | A | 5.82E-04 | 0.91 | [10] |
| rs3184504 | 12 | 110368991 | *SH2B3* | C | 1.77E-21 | 0.79 | [3] |
| rs6491500 | 13 | 98871666 | *GPR183* | G | 1.44E-05 | 1.11 | [11] |
| rs927292 | 14 | 68328594 | *14q24.1* | C | 6.78E-07 | 0.87 | [3] |
| rs2616767 | 14 | 97500135 | *14q32.2* | G | 1.73E-03 | 1.09 | [3] |
| rs941576 | 14 | 100375798 | *DLK1* | G | 9.33E-05 | 0.91 | [12] |
| rs12908309 | 15 | 36715969 | *RASGRP1* | A | 4.31E-08 | 0.85 | [13] |
| rs12148472 | 15 | 77018533 | *15q25.1* | C | 2.37E-06 | 0.83 | [3,6] |
| rs12927355 | 16 | 11102272 | *16p13.13* | T | 1.91E-16 | 0.80 | [1-3,6,7] |
| rs9924471 | 16 | 28499031 | *IL27* | A | 1.21E-11 | 1.24 | [3] |
| rs8056814 | 16 | 73809828 | *16q23.1* | A | 1.13E-07 | 1.25 | [3] |
| rs12150079 | 17 | 35278943 | *ORMDL3* | A | 3.98E-04 | 1.10 | [3] |
| rs1358174 | 17 | 36011285 | *17q21.2* | G | 1.67E-04 | 0.91 | [3] |
| rs1893217 | 18 | 12799340 | *PTPN2* | G | 1.63E-08 | 1.20 | [1,3,6,7] |
| rs1790575 | 18 | 65670059 | *CD226* | C | 9.98E-05 | 1.10 | [1] |
| rs1051738 | 19 | 10438843 | *TYK2* | A | 1.34E-05 | 0.87 | [12] |
| rs4804000 | 19 | 51968294 | *19q13.32* | A | 6.31E-08 | 0.84 | [3] |
| rs202536 | 20 | 1614253 | *20p13* | C | 1.11E-06 | 1.14 | [3] |
| rs11203203 | 21 | 42709255 | *UBASH3A* | A | 1.44E-07 | 1.14 | [3,8] |
| rs2412970 | 22 | 28816826 | *22q12.2* | G | 3.76E-04 | 0.91 | [3] |
| rs229526 | 22 | 35911368 | *IL2RB* | C | 8.33E-08 | 1.17 | [3,6] |

**References**

1. Todd JA, Walker NM, Cooper JD, Smyth DJ, Downes K, et al. (2007) Robust associations of four new chromosome regions from genome-wide analyses of type 1 diabetes. Nat Genet 39: 857-864.

2. Hakonarson H, Grant SFA, Bradfield JP, Marchand L, Kim CE, et al. (2007) A genome-wide association study identifies KIAA0350 as a type 1 diabetes gene. Nature 448: 591-594.

3. Barrett JC, Clayton DG, Concannon P, Akolkar B, Cooper JD, et al. (2009) Genome-wide association study and meta-analysis find that over 40 loci affect risk of type 1 diabetes. Nat Genet 41: 703-707.

4. Smyth DJ, Plagnol V, Walker NM, Cooper JD, Downes K, et al. (2008) Shared and distinct genetic variants in type 1 diabetes and celiac disease. N Engl J Med 359: 2767-2777.

5. Fung EY, Smyth DJ, Howson JM, Cooper JD, Walker NM, et al. (2009) Analysis of 17 autoimmune disease-associated variants in type 1 diabetes identifies 6q23/TNFAIP3 as a susceptibility locus. Genes Immun 10: 188-191.

6. Cooper JD, Smyth DJ, Smiles AM, Plagnol V, Walker NM, et al. (2008) Meta-analysis of genome-wide association study data identifies additional type 1 diabetes risk loci. Nat Genet 40: 1399-1401.

7. Wellcome Trust Case Control Consortium (2007) Genome-wide association study of 14,000 cases of seven common diseases and 3,000 shared controls. Nature 447: 661-678.

8. Grant SF, Qu HQ, Bradfield JP, Marchand L, Kim CE, et al. (2009) Follow-up analysis of genome-wide association data identifies novel loci for type 1 diabetes. Diabetes 58: 290-295.

9. Hakonarson H, Qu HQ, Bradfield JP, Marchand L, Kim CE, et al. (2008) A novel susceptibility locus for type 1 diabetes on Chr12q13 identified by a genome-wide association study. Diabetes 57: 1143-1146.

10. Bailey R, Cooper JD, Zeitels L, Smyth DJ, Yang JH, et al. (2007) Association of the vitamin D metabolism gene CYP27B1 with type 1 diabetes. Diabetes 56: 2616-2621.

11. Heinig M, Petretto E, Wallace C, Bottolo L, Rotival M, et al. A trans-acting locus regulates an anti-viral expression network and type 1 diabetes risk. Nature 467: 460-464.

12. Wallace C, Smyth DJ, Maisuria-Armer M, Walker NM, Todd JA, et al. The imprinted DLK1-MEG3 gene region on chromosome 14q32.2 alters susceptibility to type 1 diabetes. Nat Genet 42: 68-71.

13. Qu HQ, Grant SF, Bradfield JP, Kim C, Frackelton E, et al. (2009) Association of RASGRP1 with type 1 diabetes is revealed by combined follow-up of two genome-wide studies. J Med Genet 46: 553-554.
