# Supplementary material for: A Genome-Wide Meta-Analysis of Six Type 1 Diabetes Cohorts Identifies Multiple Associated Loci
Source: PLoS Genet. 2011 Sep 29;7(9):e1002293. doi: 10.1371/journal.pgen.1002293 (PMC3183083; doi:10.1371/journal.pgen.1002293)
Supplement: Table S2 — P-values and odds ratios of discovery and replication cohort are shown for all SNPs taken forward to replication stage. Combined P-values are shown for all SNPs that had the same direction of effect. P-values were combined using the Fishers combined P-value method implemented in Haploview. NA refers to a different direction of effect and the P-value was never computed. One SNP, rs722988, which failed in the genotyping assay is not shown. (DOC) [file pgen.1002293.s009.doc]

**Table S2**

|  |  |  |  | **Discovery** |  | **Replication** |  | **Combined** |
| --- | --- | --- | --- | --- | --- | --- | --- | --- |
| **SNP** | **Chr** | **Position** | **Effect Allele** | ***P*-value** | **OR** | ***P*-value** | **OR** | ***P*** |
|  |  |  |  |  |  |  |  |  |
| rs539514 | 13 | 75224283 | A | 1.74E-07 | 0.88 | 1.16E-05 | 0.70 | 5.66E-11 |
| rs478222 | 2 | 25155259 | T | 1.12E-07 | 0.87 | 1.32E-03 | 0.82 | 3.50E-09 |
| rs924043 | 6 | 170220950 | T | 1.12E-06 | 0.84 | 3.16E-04 | 0.74 | 8.06E-09 |
| rs550448 | 7 | 28195567 | G | 7.70E-06 | 0.85 | 3.29E-03 | 0.76 | 4.68E-07 |
| rs12679857 | 8 | 120046518 | G | 4.76E-06 | 0.88 | 4.71E-03 | 0.83 | 4.17E-07 |
| rs6547853 | 2 | 28500305 | A | 7.40E-06 | 0.89 | 5.67E-03 | 0.84 | 7.54E-07 |
| rs4084127 | 11 | 69305704 | A | 1.71E-06 | 0.88 | 9.23E-02 | 0.90 | 2.63E-06 |
| rs10786436 | 10 | 100290172 | T | 2.95E-07 | 1.14 | 1.53E-01 | 1.10 | 8.08E-07 |
| rs10137082 | 14 | 22909873 | T | 2.00E-06 | 0.87 | 1.89E-01 | 0.91 | 5.96E-06 |
| rs235219 | 1 | 12186942 | A | 3.92E-06 | 1.19 | 2.14E-01 | 1.13 | 1.26E-05 |
| rs2838873 | 21 | 45563781 | T | 3.79E-06 | 1.14 | 2.15E-01 | 1.10 | 1.22E-05 |
| rs4236146 | 6 | 57068571 | T | 4.71E-06 | 0.86 | 2.49E-01 | 0.92 | 1.72E-05 |
| rs10999432 | 10 | 72040730 | G | 6.74E-06 | 0.89 | 3.46E-01 | 0.94 | 3.26E-05 |
| rs17098179 | 14 | 98545127 | C | 4.04E-06 | 0.88 | 3.80E-01 | 0.94 | 2.21E-05 |
| rs11105447 | 12 | 88794561 | C | 4.23E-06 | 1.20 | 3.90E-01 | 1.09 | 2.36E-05 |
| rs7935611 | 11 | 85965655 | T | 2.87E-06 | 1.38 | 4.61E-01 | 1.12 | 1.92E-05 |
| rs2468844 | 11 | 18223603 | C | 4.83E-06 | 1.32 | 4.75E-01 | 1.07 | 3.21E-05 |
| rs1839308 | 3 | 167200111 | C | 5.40E-06 | 0.83 | 4.81E-01 | 0.94 | 3.60E-05 |
| rs4848967 | 2 | 112504889 | A | 7.63E-06 | 0.85 | 4.90E-01 | 0.94 | 5.04E-05 |
| rs7749015 | 6 | 38846515 | G | 8.52E-07 | 0.84 | 5.16E-01 | 0.95 | 6.87E-06 |
| rs602662 | 19 | 53898797 | G | 5.92E-06 | 0.89 | 5.23E-01 | 0.96 | 4.23E-05 |
| rs7857130 | 9 | 34515976 | G | 9.33E-06 | 1.12 | 6.38E-01 | 1.03 | 7.75E-05 |
| rs6681171 | 1 | 210958736 | G | 1.47E-06 | 1.18 | 6.44E-01 | 1.04 | 1.41E-05 |
| rs2269245 | 1 | 63880481 | A | 4.24E-07 | 1.16 | 6.56E-01 | 1.03 | 4.47E-06 |
| rs11195417 | 10 | 112821984 | A | 8.31E-06 | 1.18 | 6.96E-01 | 1.04 | 7.55E-05 |
| rs7420089 | 2 | 68611893 | T | 1.75E-06 | 0.68 | 9.03E-01 | 0.97 | 2.27E-05 |
| rs2160774 | 7 | 97125794 | G | 8.74E-06 | 1.25 | 9.55E-01 | 1.01 | 1.00E-04 |
| rs2212517 | 11 | 132674640 | C | 7.04E-06 | 0.80 | 1.00E+00 | 1.00 | 9.06E-05 |
| rs240650 | 8 | 15712852 | A | 9.66E-06 | 0.86 | 9.68E-01 | 1.00 | NA |
| rs11753311 | 6 | 150681353 | A | 1.27E-08 | 1.39 | 1.82E-01 | 0.85 | NA |
| rs818017 | 2 | 206610478 | A | 3.85E-07 | 1.14 | 6.11E-02 | 0.88 | NA |
| rs1544797 | 16 | 22627690 | A | 2.24E-06 | 1.14 | 3.44E-01 | 0.94 | NA |
| rs760427 | 21 | 44540419 | A | 4.54E-06 | 1.17 | 9.65E-01 | 1.00 | NA |
| rs671944 | 1 | 36067219 | C | 1.52E-08 | 1.24 | 7.84E-01 | 0.96 | NA |
| rs4894345 | 3 | 140516739 | C | 4.50E-07 | 1.16 | 6.28E-02 | 0.88 | NA |
| rs7533782 | 1 | 199099207 | G | 2.66E-06 | 1.15 | 1.38E-01 | 0.90 | NA |
| rs1867511 | 15 | 23599672 | G | 2.98E-06 | 1.14 | 3.08E-01 | 0.93 | NA |
| rs3096 | 17 | 43384431 | G | 8.97E-06 | 0.89 | 5.25E-01 | 1.04 | NA |
| rs6499137 | 16 | 66229305 | G | 2.82E-06 | 1.22 | 3.52E-02 | 0.80 | NA |
| rs6832151 | 4 | 39998408 | G | 3.21E-06 | 1.14 | 5.99E-01 | 0.96 | NA |
| rs11681201 | 2 | 204316422 | T | 6.31E-08 | 1.17 | 7.65E-01 | 0.98 | NA |
| rs4951225 | 1 | 203718178 | T | 4.53E-08 | 0.85 | 7.24E-01 | 1.03 | NA |
| rs913830 | 20 | 16172418 | T | 7.24E-06 | 1.19 | 9.33E-02 | 0.75 | NA |
| rs17268499 | 10 | 14948577 | T | 2.65E-11 | 1.33 | 1.79E-02 | 0.82 | NA |
| rs3923616 | 12 | 3088161 | T | 4.97E-06 | 1.20 | 2.28E-01 | 0.89 | NA |
| rs10196577 | 2 | 179808829 | T | 2.90E-07 | 1.30 | 4.80E-01 | 0.94 | NA |
| rs1882169 | 12 | 79781029 | T | 5.83E-06 | 1.12 | 3.68E-01 | 0.94 | NA |
| rs3821440 | 3 | 24157816 | T | 7.57E-06 | 1.16 | 4.93E-01 | 0.94 | NA |
| rs11665398 | 18 | 18559380 | T | 3.84E-06 | 1.16 | 6.63E-01 | 0.97 | NA |
| rs12507259 | 4 | 5163879 | T | 4.16E-07 | 1.18 | 9.40E-01 | 0.99 | NA |
| rs8013660 | 14 | 43601178 | T | 7.52E-06 | 1.22 | 1.07E-01 | 0.84 | NA |
| rs5755038 | 22 | 33141838 | T | 1.41E-06 | 1.18 | 4.19E-01 | 0.93 | NA |
