# Supplementary material for: A Genome-Wide Meta-Analysis of Six Type 1 Diabetes Cohorts Identifies Multiple Associated Loci
Source: PLoS Genet. 2011 Sep 29;7(9):e1002293. doi: 10.1371/journal.pgen.1002293 (PMC3183083; doi:10.1371/journal.pgen.1002293)
Supplement: Table S3 — P-values for the six SNPs highlighted in Table 1 following adjustment for lambdas. (DOC) [file pgen.1002293.s010.doc]

**Table S3**

| **SNP** | **Corrected Meta-analysis *P*-value** | **Corrected Combined *P*-value** |
| --- | --- | --- |
| rs539514 | 9.88x10^-7^ | 3.00x10^-10^ |
| rs478222 | 7.28x10^-7^ | 2.09x10^-8^ |
| rs924043 | 7.61x10^-6^ | 5.02x10^-8^ |
|  |  |  |
| rs550448 | 2.89x10^-5^ | 1.63x10^-6^ |
| rs12679857 | 2.21x10^-5^ | 1.78x10^-6^ |
| rs6547853 | 3.40x10^-5^ | 3.17x10^-6^ |
