# Supplementary material for: A Genome-Wide Meta-Analysis of Six Type 1 Diabetes Cohorts Identifies Multiple Associated Loci
Source: PLoS Genet. 2011 Sep 29;7(9):e1002293. doi: 10.1371/journal.pgen.1002293 (PMC3183083; doi:10.1371/journal.pgen.1002293)
Supplement: Text S1 — Correlation outcomes of Eigenvectors with known continental ancestry. (DOC) [file pgen.1002293.s011.doc]

**Text S1**

**Correlation of Eigenvectors with known continental ancestry:** The lamda from the Affymetrix cohort with correction is 1.11 and without correction (while still using the dummy variable for continental location) is 1.57. The lamda from the Illumina cohort with correction is 1.17 and without correction is a highly unacceptable 25.47. The only information that we had about ancestry was whether the samples were from North America or from the UK and that they were all Caucasian. We performed a correlation between each eigenvector used and continental ancestry in both the Affymetrix and Illumina cohorts. In the Illumina cohort, the 4^th^ eigenvector had a correlation coefficient of -.81 with continental ancestry and the 2^nd^ eigenvector had a correlation coefficient of -.50 with continental ancestry. All the other eigenvectors had a correlation coefficient between 0.2 and -0.2. In the Affymetrix cohort, the 1^st^ eigenvector had a correlation coefficient of -.29 with continental ancestry and the 3^rd^ eigenvector had a correlation coefficient of -.37 with continental ancestry. All the other eigenvectors had a correlation coefficient between 0.103 and -0.103. There was also the dummy variable used in the logistic regression which correlates perfectly with continental ancestry.
